# *catena*-Poly[[[(pyridine-κ*N*)copper(II)]-μ-3-{1-[(2-amino­eth­yl)imino]­eth­yl}-6-methyl-2-oxo-2*H*-pyran-4-olato-κ^4^ *N*,*N*,*O* ^4^:*O* ^2^] perchlorate]

**DOI:** 10.1107/S1600536811046411

**Published:** 2011-11-09

**Authors:** Ali Ourari, Wassila Derafa, Sofiane Bouacida, Djouhra Aggoun

**Affiliations:** a Laboratoire d’Electrochimie, d’Ingénierie Moléculaire et de Catalyse Redox (LEIMCR), Faculté des Sciences de l’Ingénieur, Université Farhat Abbas, Sétif 19000, Algeria; b Unité de Recherche de Chimie de l’Environnement et Moléculaire Structurale, CHEMS, Université Mentouri–Constantine, 25000 Algeria

## Abstract

In the title compound, {[Cu(C_10_H_13_N_2_O_3_)(C_5_H_5_N)]ClO_4_}_*n*_, the Cu^II^ atom has an N_3_O_2_ coordination sphere. The complex contains two different ligands, *viz.* a pyridine mol­ecule and a Schiff base mol­ecule, resulting from the condensation of ethyl­enodiamine with dehydro­acetic acid. The Cu^II^ atom exhibits a square-pyramidal geometry: three of the four donors of the pyramid base belong to the Schiff base ligand (an N atom from the amine group, a second N atom from the imine group and the O atom of the pyran­one residue) and the fourth donor is the pyridine N atom. The coordination around the metal ion is completed by a longer axial bond to the pyran­one O atom of an adjacent Schiff base, so forming a one-dimensional polymer. The complex has a +1 charge that is compensated by a perchlorate ion. The crystal packing, which can be described as alternating chains of cations and tetra­hedral perchlorate anions along the *a* axis, is stabilized by inter­molecular N—H⋯O, C—H⋯O and C—H⋯N hydrogen-bonding interactions.

## Related literature

For the synthesis of similar compounds: El-Abbassi *et al.* (1987 ▶); Fettouhi *et al.* (1996 ▶); El-Kihel *et al.* (1999 ▶); Tan & Kok-Peng Ang (1988 ▶); Djerrari *et al.* (2002 ▶); El-Kubaisi & Ismail (1994 ▶); Danilova *et al.* (2003 ▶); Munde *et al.* (2010 ▶). For their applications, see: Maiti *et al.* (1988 ▶); Mohan *et al.* (1981 ▶); Das & Livingstoone (1976 ▶); Moutet & Ali Ourari (1997 ▶); Ourari *et al.* (2008 ▶).
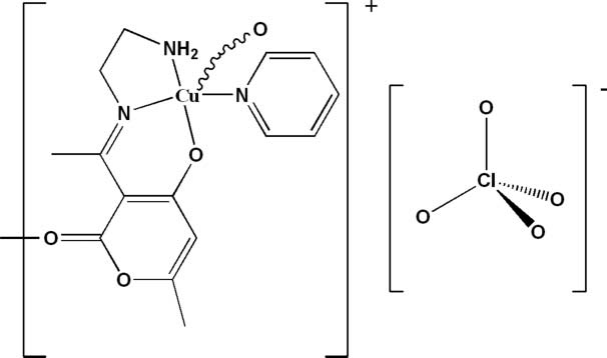


## Experimental

### 

#### Crystal data

[Cu(C_10_H_13_N_2_O_3_)(C_5_H_5_N)]ClO_4_
*M* *_r_* = 451.32
Orthorhombic, 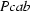

*a* = 8.8090 (2) Å
*b* = 19.9017 (4) Å
*c* = 20.9053 (5) Å
*V* = 3664.99 (14) Å^3^
*Z* = 8
Mo *K*α radiation
μ = 1.38 mm^−1^
*T* = 295 K
0.12 × 0.11 × 0.05 mm

#### Data collection

Nonius KappaCCD diffractometer
7008 measured reflections
3731 independent reflections
2619 reflections with *I* > 2σ(*I*)
*R*_int_ = 0.022

#### Refinement

*R*[*F* ^2^ > 2σ(*F* ^2^)] = 0.040
*wR*(*F* ^2^) = 0.121
*S* = 1.03
3731 reflections
246 parameters
H-atom parameters constrained
Δρ_max_ = 0.45 e Å^−3^
Δρ_min_ = −0.49 e Å^−3^

### 

Data collection: *COLLECT* (Nonius, 1998 ▶); cell refinement: *SCALEPACK* (Otwinowski & Minor, 1997 ▶); data reduction: *DENZO* (Otwinowski & Minor, 1997 ▶) and *SCALEPACK*; program(s) used to solve structure: *SIR2002* (Burla *et al.*, 2005 ▶); program(s) used to refine structure: *SHELXL97* (Sheldrick, 2008 ▶); molecular graphics: *ORTEP-3 for Windows* (Farrugia, 1997 ▶) and *DIAMOND* (Brandenburg & Berndt, 2001 ▶); software used to prepare material for publication: *WinGX* (Farrugia, 1999 ▶).

## Supplementary Material

Crystal structure: contains datablock(s) global, I. DOI: 10.1107/S1600536811046411/go2033sup1.cif

Structure factors: contains datablock(s) I. DOI: 10.1107/S1600536811046411/go2033Isup2.hkl

Additional supplementary materials:  crystallographic information; 3D view; checkCIF report

## Figures and Tables

**Table 1:** Selected bond lengths (Å)

N1—Cu1	2.049 (2)
N2—Cu1	2.001 (3)
N3—Cu1	1.974 (2)
O1—Cu1	1.914 (2)
O3—Cu1^i^	2.358 (2)

Symmetry code: (i) 
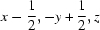
.

**Table 2:** Hydrogen-bond geometry (Å, °)

*D*—H⋯*A*	*D*—H	H⋯*A*	*D*⋯*A*	*D*—H⋯*A*
N2—H2*A*⋯O11^ii^	0.90	2.34	3.182 (4)	156
N2—H2*A*⋯O41^ii^	0.90	2.57	3.338 (4)	144
N2—H2*B*⋯O31^iii^	0.90	2.31	3.142 (4)	153
C1—H1⋯O1	0.93	2.29	2.842 (4)	118
C5—H5⋯N2	0.93	2.59	3.121 (4)	117
C8—H8*B*⋯O3	0.96	2.39	2.809 (4)	106

Symmetry codes: (ii) 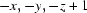; (iii) 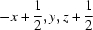.

**Fig. 1.**
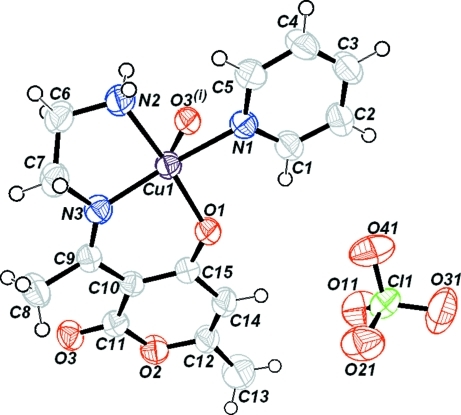
The asymmetric unit of (I) with the atom-labelling scheme. Displacement ellipsoids are drawn at the 50% probability level. H atoms are represented as small spheres of arbitrary radii.

**Fig. 2.**
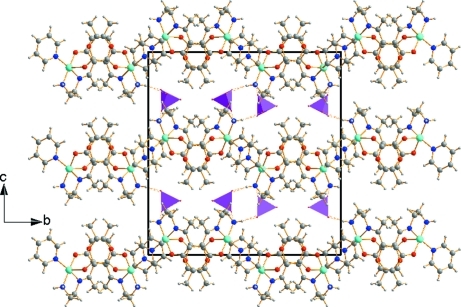
Connexion between cationic chains in zigzag with anionic tetrahedral via N—H···O hydrogen bond showing in dashed line.

## supplementary crystallographic information

### Comment

The dehydroacetic acid is a row material which is involved in the synthesis of the most heterocyclic compounds (El-Abbassi *et al.*, 1987; Fettouhi *et al.*, 1996; El-Kihel *et al.*, 1999) and the chelating agents such as the Schiff bases. These ligands are also currently applied in coordination chemistry for the synthesis of Schiff base complexes of transition metals (Tan *et al.*, 1988; El-Kubaisi *et al.*, 1994; Munde *et al.*, 2010). Additionally, it was often shown that the heterocyclic compounds resulting from this molecule exhibit some therapeutic activities (Das *et al.*, 1976; Mohan *et al.*, 1981; Maiti *et al.*, 1988) useful for the human diseases while the Schiff base complexes obtained from its ligands showed an important catalytic activity particularly in the oxidation reactions as those carried out according the cytochrome P450 model (Moutet *et al.*, 1997; Ourari *et al.*, 2008). Thus, we have attempted to synthesize the Schiff base half-units in order to use them as starting materials to obtain unsymmetrical tetradentate Schiff base complexes according the Danilova method's (Danilova *et al.*, 2003). So, we describe here the formation of a new copper Schiff base complex from dehydroacetic acid, ethylenediamine, copper perchlorate and pyridine in methanolic solution. This complex was formed in one pot with only one azomethine (–CH=N–) group yielding an unreacted amino group of ethylenediamine leading to an acceptable yield 68%. In this case, it can noted that the ring of the dehydroacetic acid seems to be not open during the reaction as it was reported in the literature (Djerrari *et al.*, 2002) in presence of nucleophile agents such as the pyridinic derivatives. This behavior may be due to an inhibition of the nucleophilic effect of the pyridine since the reaction was conducted in methanolic solution at room temperature and without reflux. Finally, the resulting compound was confirmed by crystallographic studies as further discussed.

The asymetric unit of ionic structure of (I), and the atomic numbering used, is illustrated in Fig. 1. The Cu^II^ ion is five coordinated in a square-pyramidal geometry by three N atoms of pyridine,imine and amine group and two O atom of pyranone moiety. The bond lengths for co-ordination Cu^II^ sphere is ranging from 1.974 (2) to 2.049 (2) Å for Cu-N distances and Cu-O = 1.914 (2) Å and 1.914 (2) Å (Table 2).

The crystal packing in the title structure can be described by alterning chains of cations and tetrahedral anions of perchlorate along the *c* axis (Fig. 2). It is stabilized by intermolecular N—H···O, C—H···O and C—H···N hydrogen bonding (Table 1). These interactions link the molecules within the layers and also link the layers together and reinforcing the cohesion of the ionic structure.

### Experimental

This complex was obtained by mixing stoechiometric quantities of dehydroacetic acid 0.168 g (1 mMol) with copper perchlorate 0.373 g (1 mMol) in methanol. To this mixture was added an excess of pyridine and then 0.060 g (1 mMol) of ethylenediamine dissolved as well in methanol. After two hours of reaction, a mallow precipitate was observed which is immediately recovered by filtration. It was copiously washed with methanol. Its suitable single-crystal was so obtained by slow evaporation from the filtrate.

### Refinement

The remaining H atoms were localized on Fourier maps but introduced in calculated positions and treated as riding on their parent atoms (C and N) with C—H = 0.96 Å (methyl), 0.97Å (methylene) or 0.93 Å (aromatic) and N—H = 0.90 Å with *U*_iso_(H) = 1.2*U*_eq_(C and N) or *U*_iso_(H) = 1.5*U*_eq_(methyl).

### Crystal data

**Table:**

[Cu(C_10_H_13_N_2_O_3_)(C_5_H_5_N)]ClO_4_	*D*_x_ = 1.636 Mg m^−^^3^
*M**_r_* = 451.32	Mo *K*α radiation, λ = 0.71073 Å
Orthorhombic, *P**c**a**b*	Cell parameters from 4212 reflections
*a* = 8.8090 (2) Å	θ = 1.0–26.4°
*b* = 19.9017 (4) Å	µ = 1.38 mm^−^^1^
*c* = 20.9053 (5) Å	*T* = 295 K
*V* = 3664.99 (14) Å^3^	Plate, black
*Z* = 8	0.12 × 0.11 × 0.05 mm
*F*(000) = 1848	

### Data collection

**Table:**

Nonius KappaCCD diffractometer	2619 reflections with *I* > 2σ(*I*)
Radiation source: Enraf Nonius FR590	*R*_int_ = 0.022
graphite	θ_max_ = 26.4°, θ_min_ = 3.1°
Detector resolution: 9 pixels mm^-1^	*h* = 0→10
CCD rotation images, thick slices scans	*k* = 0→24
7008 measured reflections	*l* = 0→26
3731 independent reflections	

### Refinement

**Table:**

Refinement on *F*^2^	Primary atom site location: structure-invariant direct methods
Least-squares matrix: full	Secondary atom site location: difference Fourier map
*R*[*F*^2^ > 2σ(*F*^2^)] = 0.040	Hydrogen site location: inferred from neighbouring sites
*wR*(*F*^2^) = 0.121	H-atom parameters constrained
*S* = 1.03	*w* = 1/[σ^2^(*F*_o_^2^) + (0.0723*P*)^2^ + 0.807*P*] where *P* = (*F*_o_^2^ + 2*F*_c_^2^)/3
3731 reflections	(Δ/σ)_max_ = 0.001
246 parameters	Δρ_max_ = 0.45 e Å^−^^3^
0 restraints	Δρ_min_ = −0.49 e Å^−^^3^

### Special details

**Table:**

Geometry. All e.s.d.'s (except the e.s.d. in the dihedral angle between two l.s. planes) are estimated using the full covariance matrix. The cell e.s.d.'s are taken into account individually in the estimation of e.s.d.'s in distances, angles and torsion angles; correlations between e.s.d.'s in cell parameters are only used when they are defined by crystal symmetry. An approximate (isotropic) treatment of cell e.s.d.'s is used for estimating e.s.d.'s involving l.s. planes.
Refinement. Refinement of *F*^2^ against ALL reflections. The weighted *R*-factor *wR* and goodness of fit *S* are based on *F*^2^, conventional *R*-factors *R* are based on *F*, with *F* set to zero for negative *F*^2^. The threshold expression of *F*^2^ > σ(*F*^2^) is used only for calculating *R*-factors(gt) *etc*. and is not relevant to the choice of reflections for refinement. *R*-factors based on *F*^2^ are statistically about twice as large as those based on *F*, and *R*- factors based on ALL data will be even larger.

### Fractional atomic coordinates and isotropic or equivalent isotropic displacement parameters (Å^2^)

**Table:**

	*x*	*y*	*z*	*U*_iso_*/*U*_eq_	
C1	0.2273 (4)	0.02862 (16)	0.45790 (16)	0.0517 (8)	
H1	0.1798	0.0658	0.4399	0.062*	
C2	0.3060 (4)	−0.01413 (18)	0.41827 (18)	0.0600 (9)	
H2	0.311	−0.0058	0.3746	0.072*	
C3	0.3770 (4)	−0.06940 (19)	0.4441 (2)	0.0600 (9)	
H3	0.4319	−0.0988	0.4183	0.072*	
C4	0.3652 (4)	−0.08009 (18)	0.5081 (2)	0.0630 (10)	
H4	0.4119	−0.1171	0.5268	0.076*	
C5	0.2830 (4)	−0.03545 (16)	0.54523 (18)	0.0560 (8)	
H5	0.2742	−0.0439	0.5888	0.067*	
C6	0.1257 (4)	0.09736 (17)	0.71225 (16)	0.0559 (9)	
H6A	0.1883	0.137	0.7179	0.067*	
H6B	0.1258	0.0722	0.752	0.067*	
C7	−0.0333 (4)	0.11746 (18)	0.69521 (16)	0.0567 (9)	
H7A	−0.1003	0.0789	0.6975	0.068*	
H7B	−0.0699	0.1514	0.7247	0.068*	
C8	−0.2344 (4)	0.22163 (17)	0.65805 (17)	0.0576 (9)	
H8A	−0.2762	0.1859	0.6834	0.086*	
H8B	−0.3139	0.2426	0.6338	0.086*	
H8C	−0.1879	0.2543	0.6856	0.086*	
C9	−0.1166 (3)	0.19342 (14)	0.61287 (14)	0.0398 (6)	
C10	−0.1085 (3)	0.21966 (14)	0.54728 (14)	0.0380 (6)	
C11	−0.1603 (3)	0.28666 (15)	0.53460 (15)	0.0430 (7)	
C12	−0.1244 (4)	0.26748 (18)	0.42205 (14)	0.0508 (8)	
C13	−0.1460 (6)	0.3012 (2)	0.3588 (2)	0.0908 (15)	
H13A	−0.0797	0.3394	0.3559	0.136*	
H13B	−0.2495	0.3158	0.3549	0.136*	
H13C	−0.1228	0.2702	0.3251	0.136*	
C14	−0.0700 (4)	0.20627 (18)	0.43217 (15)	0.0562 (9)	
H14	−0.0417	0.1798	0.3975	0.067*	
C15	−0.0541 (3)	0.18023 (15)	0.49603 (14)	0.0423 (7)	
N1	0.2158 (3)	0.01933 (12)	0.52137 (12)	0.0432 (6)	
N2	0.1869 (3)	0.05540 (13)	0.65998 (12)	0.0532 (7)	
H2A	0.1582	0.0124	0.6655	0.064*	
H2B	0.289	0.057	0.6602	0.064*	
N3	−0.0300 (3)	0.14435 (12)	0.62977 (12)	0.0429 (6)	
O1	0.0066 (2)	0.12212 (10)	0.50150 (10)	0.0491 (5)	
O2	−0.1684 (3)	0.30769 (10)	0.47159 (11)	0.0541 (6)	
O3	−0.1950 (3)	0.33001 (10)	0.57362 (11)	0.0519 (6)	
O11	−0.1778 (3)	0.09728 (16)	0.29504 (16)	0.0869 (9)	
O21	0.0241 (4)	0.16739 (14)	0.26635 (16)	0.0868 (9)	
O31	−0.0210 (4)	0.06891 (19)	0.21098 (16)	0.1067 (11)	
O41	0.0701 (4)	0.06507 (16)	0.31535 (17)	0.0910 (10)	
Cl1	−0.02517 (10)	0.09943 (4)	0.27165 (4)	0.0553 (2)	
Cu1	0.10791 (4)	0.089862 (17)	0.576431 (17)	0.03931 (14)	

### Atomic displacement parameters (Å^2^)

**Table:**

	*U*^11^	*U*^22^	*U*^33^	*U*^12^	*U*^13^	*U*^23^
C1	0.057 (2)	0.0475 (18)	0.0504 (19)	0.0085 (15)	0.0026 (16)	−0.0018 (15)
C2	0.065 (2)	0.062 (2)	0.053 (2)	0.0106 (18)	0.0082 (17)	−0.0093 (16)
C3	0.054 (2)	0.057 (2)	0.069 (2)	0.0124 (16)	0.0062 (17)	−0.0149 (19)
C4	0.063 (2)	0.0489 (19)	0.078 (3)	0.0197 (16)	−0.0030 (19)	−0.0071 (18)
C5	0.064 (2)	0.0480 (18)	0.056 (2)	0.0109 (16)	−0.0041 (17)	−0.0006 (16)
C6	0.075 (2)	0.0528 (19)	0.0397 (18)	0.0080 (17)	−0.0046 (16)	0.0028 (14)
C7	0.070 (2)	0.062 (2)	0.0386 (18)	0.0066 (17)	0.0109 (16)	0.0073 (16)
C8	0.065 (2)	0.0550 (19)	0.053 (2)	0.0112 (17)	0.0181 (17)	0.0024 (16)
C9	0.0388 (15)	0.0399 (15)	0.0406 (16)	−0.0040 (12)	0.0029 (12)	−0.0041 (12)
C10	0.0381 (15)	0.0365 (14)	0.0392 (15)	0.0007 (12)	−0.0003 (12)	0.0003 (12)
C11	0.0422 (16)	0.0434 (16)	0.0434 (17)	−0.0011 (13)	0.0003 (13)	0.0008 (13)
C12	0.062 (2)	0.0546 (19)	0.0354 (17)	0.0125 (15)	0.0016 (14)	0.0023 (14)
C13	0.123 (4)	0.094 (3)	0.055 (3)	0.040 (3)	0.002 (2)	0.021 (2)
C14	0.073 (2)	0.061 (2)	0.0352 (17)	0.0186 (17)	−0.0016 (15)	−0.0023 (14)
C15	0.0423 (16)	0.0458 (16)	0.0387 (16)	0.0055 (13)	−0.0009 (12)	−0.0036 (13)
N1	0.0465 (14)	0.0377 (12)	0.0456 (15)	0.0032 (10)	−0.0024 (11)	−0.0017 (11)
N2	0.0679 (18)	0.0489 (15)	0.0428 (15)	0.0100 (13)	−0.0001 (13)	0.0044 (12)
N3	0.0466 (15)	0.0428 (13)	0.0394 (14)	0.0003 (11)	0.0031 (11)	0.0037 (11)
O1	0.0591 (13)	0.0467 (12)	0.0415 (12)	0.0163 (10)	−0.0048 (10)	−0.0058 (9)
O2	0.0671 (14)	0.0458 (12)	0.0494 (13)	0.0110 (11)	0.0009 (11)	0.0060 (10)
O3	0.0640 (14)	0.0388 (11)	0.0528 (13)	0.0068 (10)	0.0018 (11)	−0.0062 (10)
O11	0.0555 (15)	0.116 (2)	0.089 (2)	−0.0077 (16)	0.0063 (16)	0.0173 (18)
O21	0.093 (2)	0.0626 (17)	0.105 (2)	−0.0126 (16)	−0.0017 (18)	0.0161 (16)
O31	0.109 (3)	0.145 (3)	0.067 (2)	−0.022 (2)	−0.0035 (19)	−0.038 (2)
O41	0.088 (2)	0.091 (2)	0.094 (2)	0.0057 (18)	−0.0299 (18)	0.0290 (18)
Cl1	0.0575 (5)	0.0638 (5)	0.0447 (5)	−0.0077 (4)	−0.0079 (4)	0.0055 (4)
Cu1	0.0458 (2)	0.0370 (2)	0.0352 (2)	0.00416 (15)	0.00067 (15)	0.00081 (14)

### Geometric parameters (Å, °)

**Table:**

C1—N1	1.344 (4)	C10—C11	1.434 (4)
C1—C2	1.375 (4)	C11—O3	1.226 (4)
C1—H1	0.93	C11—O2	1.384 (4)
C2—C3	1.376 (5)	C12—C14	1.326 (5)
C2—H2	0.93	C12—O2	1.365 (4)
C3—C4	1.358 (6)	C12—C13	1.495 (5)
C3—H3	0.93	C13—H13A	0.96
C4—C5	1.385 (5)	C13—H13B	0.96
C4—H4	0.93	C13—H13C	0.96
C5—N1	1.337 (4)	C14—C15	1.439 (4)
C5—H5	0.93	C14—H14	0.93
C6—N2	1.477 (4)	C15—O1	1.279 (4)
C6—C7	1.499 (5)	N1—Cu1	2.049 (2)
C6—H6A	0.97	N2—Cu1	2.001 (3)
C6—H6B	0.97	N2—H2A	0.9
C7—N3	1.469 (4)	N2—H2B	0.9
C7—H7A	0.97	N3—Cu1	1.974 (2)
C7—H7B	0.97	O1—Cu1	1.914 (2)
C8—C9	1.512 (4)	O3—Cu1^i^	2.358 (2)
C8—H8A	0.96	O11—Cl1	1.431 (3)
C8—H8B	0.96	O21—Cl1	1.425 (3)
C8—H8C	0.96	O31—Cl1	1.407 (3)
C9—N3	1.288 (4)	O41—Cl1	1.417 (3)
C9—C10	1.469 (4)	Cu1—O3^ii^	2.358 (2)
C10—C15	1.412 (4)		
			
N1—C1—C2	123.2 (3)	O2—C12—C13	111.8 (3)
N1—C1—H1	118.4	C12—C13—H13A	109.5
C2—C1—H1	118.4	C12—C13—H13B	109.5
C1—C2—C3	119.2 (4)	H13A—C13—H13B	109.5
C1—C2—H2	120.4	C12—C13—H13C	109.5
C3—C2—H2	120.4	H13A—C13—H13C	109.5
C4—C3—C2	118.5 (3)	H13B—C13—H13C	109.5
C4—C3—H3	120.8	C12—C14—C15	120.9 (3)
C2—C3—H3	120.8	C12—C14—H14	119.5
C3—C4—C5	119.5 (3)	C15—C14—H14	119.5
C3—C4—H4	120.3	O1—C15—C10	125.2 (3)
C5—C4—H4	120.3	O1—C15—C14	116.7 (3)
N1—C5—C4	123.1 (3)	C10—C15—C14	118.1 (3)
N1—C5—H5	118.5	C5—N1—C1	116.6 (3)
C4—C5—H5	118.5	C5—N1—Cu1	123.7 (2)
N2—C6—C7	108.4 (3)	C1—N1—Cu1	119.7 (2)
N2—C6—H6A	110	C6—N2—Cu1	108.96 (19)
C7—C6—H6A	110	C6—N2—H2A	109.9
N2—C6—H6B	110	Cu1—N2—H2A	109.9
C7—C6—H6B	110	C6—N2—H2B	109.9
H6A—C6—H6B	108.4	Cu1—N2—H2B	109.9
N3—C7—C6	107.5 (3)	H2A—N2—H2B	108.3
N3—C7—H7A	110.2	C9—N3—C7	121.3 (3)
C6—C7—H7A	110.2	C9—N3—Cu1	128.7 (2)
N3—C7—H7B	110.2	C7—N3—Cu1	109.75 (19)
C6—C7—H7B	110.2	C15—O1—Cu1	124.85 (19)
H7A—C7—H7B	108.5	C12—O2—C11	122.0 (2)
C9—C8—H8A	109.5	C11—O3—Cu1^i^	132.6 (2)
C9—C8—H8B	109.5	O31—Cl1—O41	110.9 (2)
H8A—C8—H8B	109.5	O31—Cl1—O21	109.4 (2)
C9—C8—H8C	109.5	O41—Cl1—O21	109.14 (19)
H8A—C8—H8C	109.5	O31—Cl1—O11	108.7 (2)
H8B—C8—H8C	109.5	O41—Cl1—O11	108.8 (2)
N3—C9—C10	119.8 (3)	O21—Cl1—O11	109.94 (19)
N3—C9—C8	121.1 (3)	O1—Cu1—N3	89.50 (9)
C10—C9—C8	119.0 (3)	O1—Cu1—N2	172.52 (11)
C15—C10—C11	119.0 (3)	N3—Cu1—N2	84.80 (10)
C15—C10—C9	121.8 (3)	O1—Cu1—N1	89.20 (9)
C11—C10—C9	119.2 (3)	N3—Cu1—N1	168.32 (10)
O3—C11—O2	114.0 (3)	N2—Cu1—N1	95.41 (10)
O3—C11—C10	127.6 (3)	O1—Cu1—O3^ii^	95.50 (9)
O2—C11—C10	118.3 (3)	N3—Cu1—O3^ii^	95.48 (9)
C14—C12—O2	121.4 (3)	N2—Cu1—O3^ii^	89.86 (10)
C14—C12—C13	126.9 (3)	N1—Cu1—O3^ii^	96.20 (9)
			
N1—C1—C2—C3	0.0 (5)	C6—C7—N3—Cu1	39.9 (3)
C1—C2—C3—C4	0.8 (6)	C10—C15—O1—Cu1	−25.3 (4)
C2—C3—C4—C5	−0.1 (6)	C14—C15—O1—Cu1	155.8 (2)
C3—C4—C5—N1	−1.3 (6)	C14—C12—O2—C11	−0.7 (5)
N2—C6—C7—N3	−49.7 (4)	C13—C12—O2—C11	179.3 (3)
N3—C9—C10—C15	23.6 (4)	O3—C11—O2—C12	175.7 (3)
C8—C9—C10—C15	−152.3 (3)	C10—C11—O2—C12	−2.1 (4)
N3—C9—C10—C11	−157.8 (3)	O2—C11—O3—Cu1^i^	42.7 (4)
C8—C9—C10—C11	26.3 (4)	C10—C11—O3—Cu1^i^	−139.8 (3)
C15—C10—C11—O3	−171.4 (3)	C15—O1—Cu1—N3	31.6 (3)
C9—C10—C11—O3	10.0 (5)	C15—O1—Cu1—N1	−160.0 (3)
C15—C10—C11—O2	6.1 (4)	C15—O1—Cu1—O3^ii^	−63.8 (3)
C9—C10—C11—O2	−172.5 (2)	C9—N3—Cu1—O1	−16.1 (3)
O2—C12—C14—C15	−0.6 (6)	C7—N3—Cu1—O1	158.9 (2)
C13—C12—C14—C15	179.5 (4)	C9—N3—Cu1—N2	168.7 (3)
C11—C10—C15—O1	173.9 (3)	C7—N3—Cu1—N2	−16.2 (2)
C9—C10—C15—O1	−7.5 (5)	C9—N3—Cu1—N1	−99.8 (5)
C11—C10—C15—C14	−7.2 (4)	C7—N3—Cu1—N1	75.3 (6)
C9—C10—C15—C14	171.3 (3)	C9—N3—Cu1—O3^ii^	79.4 (3)
C12—C14—C15—O1	−176.5 (3)	C7—N3—Cu1—O3^ii^	−105.6 (2)
C12—C14—C15—C10	4.6 (5)	C6—N2—Cu1—N3	−11.2 (2)
C4—C5—N1—C1	2.0 (5)	C6—N2—Cu1—N1	−179.5 (2)
C4—C5—N1—Cu1	−175.0 (3)	C6—N2—Cu1—O3^ii^	84.3 (2)
C2—C1—N1—C5	−1.3 (5)	C5—N1—Cu1—O1	−162.4 (3)
C2—C1—N1—Cu1	175.8 (3)	C1—N1—Cu1—O1	20.7 (2)
C7—C6—N2—Cu1	36.0 (3)	C5—N1—Cu1—N3	−78.7 (6)
C10—C9—N3—C7	179.1 (3)	C1—N1—Cu1—N3	104.4 (5)
C8—C9—N3—C7	−5.1 (4)	C5—N1—Cu1—N2	11.7 (3)
C10—C9—N3—Cu1	−6.4 (4)	C1—N1—Cu1—N2	−165.2 (2)
C8—C9—N3—Cu1	169.4 (2)	C5—N1—Cu1—O3^ii^	102.2 (3)
C6—C7—N3—C9	−144.6 (3)	C1—N1—Cu1—O3^ii^	−74.7 (2)

Symmetry codes: (i) *x*−1/2, −*y*+1/2, *z*; (ii) *x*+1/2, −*y*+1/2, *z*.

### Hydrogen-bond geometry (Å, °)

**Table:**

*D*—H···*A*	*D*—H	H···*A*	*D*···*A*	*D*—H···*A*
N2—H2A···O11^iii^	0.90	2.34	3.182 (4)	156.
N2—H2A···O41^iii^	0.90	2.57	3.338 (4)	144.
N2—H2B···O31^iv^	0.90	2.31	3.142 (4)	153.
C1—H1···O1	0.93	2.29	2.842 (4)	118.
C5—H5···N2	0.93	2.59	3.121 (4)	117.
C8—H8B···O3	0.96	2.39	2.809 (4)	106.

Symmetry codes: (iii) −*x*, −*y*, −*z*+1; (iv) −*x*+1/2, *y*, *z*+1/2.
